# A scoping review of clinical reasoning research with Asian healthcare professionals

**DOI:** 10.1007/s10459-021-10060-z

**Published:** 2021-07-12

**Authors:** Ching-Yi Lee, Chang-Chyi Jenq, Madawa Chandratilake, Julie Chen, Mi-Mi Chen, Hiroshi Nishigori, Gohar Wajid, Pai-Hsuang Yang, Muhamad Saiful Bahri Yusoff, Lynn Monrouxe

**Affiliations:** 1 Department of Neurosurgery, Chang Gung Memorial Hospital Linkou Medical Center and Chang Gung University College of Medicine, Taoyuan, Taiwan; 2 Medical Education Research Center, Chang Gung Memorial Hospital Linkou Medical Center and Chang Gung University College of Medicine, Taoyuan, Taiwan; 3 Department of Nephrology, Chang Gung Memorial Hospital Linkou Medical Center and Chang Gung University College of Medicine, Taoyuan, Taiwan; 4 Department of Medical Education, Faculty of Medicine, University of Kelaniya, Kelaniya, Taiwan; 5 Department of Family Medicine and Primary Care (FMPC) and Bau Institute of Medical and Health Sciences Education (BIMHSE), The University of Hong Kong, Hong Kong, China; 6 Center for Medical Education, Graduate School of Medicine, Nagoya University, Nagoya, China; 7 World Health Organization, Geneva, Switzerland; 8 Department of Medical Education, School of Medical Sciences, Universiti Sains Malaysia, Gelugor, Malaysia; 9 School of Health Sciences, Faculty of Medicine and Health, The University of Sydney, Level 7, Susan Wakil Health Building D18, Sydney, NSW 2006 Australia

**Keywords:** Scoping review, Asia, Clinical reasoning, Health professions

## Abstract

Clinical reasoning is the thought process that guides practice. Although a plethora of clinical reasoning studies in healthcare professionals exists, the majority appear to originate from Western cultures. A scoping review was undertaken to examine clinical reasoning related research across Asian cultures. PubMed, SciVerse Scopus, Web of Science and Airiti Library databases were searched. Inclusion criteria included full-text articles published in Asian countries (2007 to 2019). Search terms included clinical reasoning, thinking process, differential diagnosis, decision making, problem-based learning, critical thinking, healthcare profession, institution, medical students and nursing students. After applying exclusion criteria, n = 240 were included in the review. The number of publications increased in 2012 (from 5%, n = 13 in 2011 to 9%, n = 22) with a steady increase onwards to 12% (n = 29) in 2016. South Korea published the most articles (19%, n = 46) followed by Iran (17%, n = 41). Nurse Education Today published 11% of the articles (n = 26), followed by BMC Medical Education (5%, n = 13). Nursing and Medical students account for the largest population groups studied. Analysis of the articles resulted in seven themes: Evaluation of existing courses (30%, n = 73) being the most frequently identified theme. Only seven comparative articles showed cultural implications, but none provided direct evidence of the impact of culture on clinical reasoning. We illuminate the potential necessity of further research in clinical reasoning, specifically with a focus on how clinical reasoning is affected by national culture. A better understanding of current clinical reasoning research in Asian cultures may assist curricula developers in establishing a culturally appropriate learning environment.

**Supplementary Information:**

The online version contains supplementary material available at 10.1007/s10459-021-10060-z.

## Introduction

Clinical reasoning, a central component of healthcare professionals’ competence, comprises the thought process that guides medical practice (Eva, 2005; Rogers, 1983). Clinical reasoning includes a range of critical thinking, judgment, decision making and problem-solving skills (Ajjawi & Higgs, 2008; Eva, 2005; Griffits, Hines, Moloney, & Ralph, 2017; Gummesson, Sundén, & Fex, 2018; Norman, 2005; Rogers, 1983). Learning how to reason can be viewed as a thinking process prone to ‘disposition’, since clinical reasoning can be influenced by personal attitudes, cultural perspectives and preconceptions (McCarthy, 2003; Scheffer & Rubenfeld, 2000). Despite clinical reasoning being culturally influenced, research investigating it across Asian settings appears to be scarce. Indeed, a recent systematic review of 58 articles (2000–2015) on teaching and assessing clinical reasoning for enhancing professional competence uncovered no research of Asian origin, and merely focused on Western models of teaching and assessing clinical reasoning (Guraya, 2016).

## Research in clinical reasoning

Over the past decades, the focus of research in clinical reasoning has shifted from understanding the process and factors associated with clinical reasoning to the subject of mental representations relating to expert knowledge (Norman, 2005). There is also a growing body of literature that has explored strategies of teaching or assessment in clinical reasoning. The hypothetic-deductive model, pattern recognition and dual process reasoning model are the most frequently identified strategies; while case studies with simulation, direct observation, script concordance tests and think aloud exams are the most commonly addressed assessment methods (Gruppen, 2017; Yazdani, Hosseinzadeh, & Hosseini, 2017). Furthermore, it has been proposed that diagnostic errors may arise from flaws in the thinking processes during clinical reasoning (Norman et al., 2017). Research has highlighted the strategies employed in the training of thinking processes that might improve clinical reasoning and decision making, hence leading to better quality in healthcare (Griffits et al., 2017; Gummesson et al., 2018; Lambe, O'Reilly, Kelly, & Curristan, 2016; Schmidt & Mamede, 2015).

In terms of cultural differences in clinical reasoning, literature is scarce. One mixed methods study examined differences in clinical reasoning of students from Asian (Indonesian) and Western (Australian) medical education settings (Findyartini, Hawthorne, McColl, & Chiavaroli, 2016). The study found that Western students appeared to be more independent self-learners, but their Asian counterparts tended to be more teacher-dependent; Western students and their tutors asserted a belief that classroom problem-based learning was sufficient for learning of clinical reasoning skills, but Asian students and tutors tended to believe them to be developed mainly in clinical settings. However, clinical reasoning in this study was defined apriori as an Analytic process. As such, the comparison of thinking processes between the two cultures was not examined.

### Culture and cognition: analytic vs Holistic thinking

Understanding the influence of culture on clinical reasoning is a key issue given today’s globalisation of healthcare education which tends to adopt Western approaches as the default (Hodges, Maniate, Martimianakis, Alsuwaidan, & Segouin, 2009). However, culture does influence thought, which in turn impacts on reasoning (including reasoning clinically). Broadly speaking, different cultures appear to favor different styles of thinking (Nakamura, 1985). Note, the theoretical basis of cross-cultural studies are underpinned by the concept of national context effects (Straus, 2009) and as such are focused at the level of the group—not the individual (i.e. individual differences within cultures are specifically acknowledged).

In terms of thinking more broadly (i.e. general cognition), according to Nisbett and his colleagues, Eastern cultures tend to favour an ‘Holistic’ approach to thinking, conversely Western cultures tend to favour an ‘Analytic’ approach (Nisbett & Masuda, 2003; Nisbett, Peng, Choi, & Norenzayan, 2001). Indeed, some cognitive differences have been observed between Analytic and Holistic ways of thinking in research on general cognition which have implications for culture, clinical reasoning and decision making (Ji, Peng, & Nisbett, 2000; Nisbett & Miyamoto, 2005; Nisbett et al., 2001).

Based on this premise, using an East Asian sample to represent Holistic thinkers and a Western sample to represent Analytic thinkers, several differences between Analytic and Holistic thinkers have been evidenced: Analytic thinkers’ confidence and performance in a cognitive task significantly increases when a sense of control is induced, but this is not the case for Holistic thinkers; Analytic thinkers tend to explain behavior concerning internal attributes, whereas Holistic thinkers tend to explain behavior regarding the interaction between internal attributes and situational factors; Analytic thinkers predominately use deterministic rules to categorise objects, whereas Holistic thinkers prefer to draw on similarities and relationships amongst objects; Analytic thinkers tend to make choices between opposing positions, whereas Holistic thinkers tend to avoid conflicts and find a compromise solution between opposing positions; Analytic thinkers tend to have a linear perspective towards the future, however, Holistic thinkers prefer a more dialectical perspective, believing that certain trends will change in the future (Choi, Koo, & Jong An, 2007; Nisbett & Masuda, 2003; Nisbett et al., 2001).

Due to the unique characteristics of each style of thinking, it has been argued that decision making across Eastern and Western cultural contexts differs (Li, Masuda, Hamamura, & Ishii, 2018). For example, studies have provided evidence about the varying amounts of information decision-makers gather before their final decisions, with some studies linking it to Analytic or Holistic styles of thinking (Choi, Choi, & Norenzayan, 2008; Choi et al., 2007; Li et al., 2018; Nisbett & Miyamoto, 2005). Thus, by contrast to Holistic thinkers, it has been argued that Analytic thinkers assume that information in every event can be understood in isolation from the whole. Alternatively, Holistic thinkers tend to consider many aspects of information before coming to a decision (Choi, Dalal, Kim-Prieto, & Park, 2003; Choi et al., 2007, 2008; Levett-Jones et al., 2010). Analytic and Holistic thinkers can also differ when deciding on the *type* of information considered to be essential (Nisbett & Masuda, 2003). In combining information, Holistic thinkers tend to seek a compromise between conflicting pieces of information, while Analytic thinkers tend to make a principal choice by carefully scrutinising each piece of information (Choi et al., 2003, 2007; Nisbett et al., 2001). We can therefore anticipate that the clinical reasoning of health professionals from Western and Eastern contexts might be dissimilar to one another.

### The scoping review

Scoping reviews are the appropriate review methodology for assessing and understanding the *extent* of knowledge in a field when no previous review has been undertaken (Peters et al., 2020). This scoping review therefore aims to address the current gap in the literature by focusing on clinical reasoning research that has been published arising from Asian cultures. In doing so, we aim to ascertain the extent to which clinical reasoning has been the focus research in Asia, including: which countries have contributed to this literature, which healthcare professions have been studied, how clinical reasoning has been conceptualized, what aspects of clinical reasoning are the focus of interest (e.g. teaching evaluation, assessment, cultural differences), where studies have been published and the funding status of the research. Such a scoping of the literature will identify key gaps alongside issues such as research rigour.

## Methods

With the Arksey and O'Malley framework (Arksey & O'Malley, 2005) and the recommendations by Levac and Peters (Levac, Colquhoun, & O'Brien, 2010; Peters et al., 2020), our scoping review approach involves the following steps: (1) scoping review questions; (2) search strategy; (3) study screening and selection; (4) data extraction; and (5) analysis and presentation of results (Arksey & O'Malley, 2005; Hidalgo et al., 2011; Khalil et al., 2016). We chose not to include the optional step of consultation with practitioners and consumers prior to developing the protocol for a number of reasons, including: (1) our research team comprise key international personnel who are also practitioners and consumers of this work across Asia; (2) the restricted timescale in which we had to develop the protocol; and (3) the financial logistics in gathering an appropriate international consultation group, running the sessions and post-session work.

### Review questions

Initially, we began with the broad research question: “What is the current status of clinical reasoning research arising from Asian cultures?”. Following a team discussion, the following specific research questions (RQs) were developed:

- RQ1: How many clinical reasoning articles have been published from an Asian origin between 2007 and 2019?
- RQ2: Which Asian countries have researched clinical reasoning?
- RQ3: Which healthcare professionals have been studied?
- RQ4: What types of studies have been undertaken (methodologies)?
- RQ5: What is the main focus of the published studies?
- RQ6: What does the literature tell us about cultural differences and similarities?
- RQ7: In what journals have the studies been published, in what language, and what is the indexing status of those journals?
- RQ8: What is the funding status of the studies?
- RQ9: Are there any trends across RQs 1–7 over time?

The rationales for these questions are as follows. Firstly, given the lack of Asian studies cited in the clinical reasoning literature, we wished to understand the prevalence and origin of studies (RQs 1 & 2). An understanding of the healthcare professions included in the literature and the types of studies enables us to better describe the landscape of the literature (RQs 3 & 4). The focus of the studies enables us to ascertain the gaps in the literature (RQ5). Specifically, we will investigate the extent to which culture has been examined across clinical reasoning studies (RQ 6). We are also interested in where articles are published and the extent to which studies on clinical reasoning across Asia are funded. This provides us with proxy information about how this research is valued (e.g. whether it is considered worthy of being funded and published in quality journals: RQs 7–8). Finally, examining trends across all these aspects over time enables us to gain a deeper understanding of the general state of clinical reasoning across Asia (e.g. is it more prevalent now? is it funded more now? what are the key issues in the literature over time? RQ 9).

### Defining the scope

Research on clinical reasoning is fragmented due to the range of definitions, conceptualisations and terminology that have been variously used (Young et al., 2018). Indeed, we note from our own discussions whilst developing inclusion and exclusion criteria for this study, that the terms clinical reasoning, critical thinking and clinical decision making have overlapping elements. We also note that some professional domains have a preference for one term over another. For example, literature in medicine has a tendency to use the term *clinical reasoning* whereas the nursing literature tends to use the term *critical thinking*. Furthermore, both of these terms include an element of clinical *decision making*. Indeed, according to Simmons (2010), *clinical reasoning* is ‘‘a complex cognitive process that uses formal and informal thinking strategies to gather and analyze patient information, evaluate the significance of this information and weigh alternative actions’’. It is the sum of decision making and critical thinking processes (Harris 1993). Given that the terms decision making, critical thinking, and clinical reasoning have fuzzy boundaries (Young et al., 2018), with decision making and critical thinking sometimes being used as sub-processes within clinical reasoning, and with clinical reasoning and critical thinking sometimes being used interchangeably across the healthcare professions education literature, we decided to include them all in our search strategy. Furthermore, because we are interested in the range of sub-processes within clinical reasoning, we were also open to these processes within the healthcare professions education literature.

### Search strategy

Four electronic databases were utilized: PubMed, SciVerse Scopus, Web of Science and Airiti Library. These were used to capture a wide range of possible articles including those published in non-English language (e.g. Airiti holds a large collection of Chinese language academic publications). Following the database searches, snowballing through hand searching reference lists results in a total of 11 additional articles. We undertook two searches across a 13-year period: 2007–2019 (initially, 15 October 2017 for the 9-year period 2007–2016, and updated on 09 March 2020 to include 2017–2019). The search terms included clinical reasoning, thinking process, differential diagnosis, decision making, problem-based learning, and critical thinking, combined with healthcare profession or institution, medical or nursing students, and trainees or residents (see online Supplement Table 1 for the full strategy). The major search strings used were (“clinical reasoning” OR “thinking process” OR “differential diagnosis” OR “decision making” OR “problem-based learning” OR “critical thinking”) AND (“healthcare profession” OR “institution” OR “medical students” OR “nursing students” OR “trainees” OR “residents”).

### Screening

All articles were collected and stored using EndNote® to eliminate duplicates. The title and abstract of citations were screened independently by two reviewers. Articles retrieved were included in the review if they met the following criteria: (a) Original full-text articles focused on clinical reasoning or its derivatives (b) Peer-reviewed articles published across the years 2007 and 2019 (c) Studies with country of publications from the list of 50 Asian countries ("United Nations geoscheme for Asia," 2021). Decisions regarding Asian countries were relatively straightforward, except for those originating from Russia, Kazakhstan, Azerbaijan, Georgia, and Turkey, as these states are in both continents of Asia and Europe. For these countries we consulted a map to determine which side of the boundary they were in before making a decision to include or exclude them. The country of publication for each article was then determined by the first author’s affiliation. Team members agreed that the start date of 2007 for inclusion should be used because it is a key transitional point in medical education in Taiwan, a leader in medical education research more generally in Asia due to its’ unique funding structure (Monrouxe, Liu, Yau, & Babovic, 2020).

The full inclusion/exclusion criteria were as follows:

- *Inclusion*: peer-reviewed full journal articles, original data, from 2007-2019, publications with at least one researcher from an Asian country, full-text available and focuses on clinical reasoning (or its derivatives);
- *Exclusion*: non-peer-reviewed articles, conference abstracts, letters, articles without full-text available or published by researchers outside of Asia.

### Data extraction

After screening, all relevant articles were imported to ATLAS.ti™ from EndNote®. A set of codes for charting content was developed to extract study characteristics required to answer our research questions such as publication year, journal type, study locations, methodology, participants and funding resources. This charting process was reviewed and pretested by the research team before implementation.

For many codes, the category is obvious (e.g. publication year, journal) but for the content codes (RQ5) relating to the focus of the studies, we undertook a thematic analysis. All authors independently read a subset of the articles to develop themes. We then met to develop an initial coding framework. Included in this framework were specific codes for the key terms of clinical reasoning, critical thinking and decision making. This enabled us to clarify the specific focus of each article as we used the authors’ terminology. Coding the data in this way meant that we could include all articles, even those that did not clearly define the specific construct of study. Further meetings throughout the process enabled us to resolve conflicts and ensure consistency across the coding. Each article was coded to only one main content theme for RQ5, although sometimes more than one could have been possible. Our rationale was to enable us to examine trends over time, so we coded to the main focus of the article, rather than having multiple coding for some articles.

### Analysis and presentation of results

The intention of a scoping review is to present an overview comprising basic descriptive analyses (e.g. frequency counts) of extracted data, rather than synthesizing results or codings of the included studies (Peters et al., 2020). We therefore use article ‘demographic’ coding (e.g. year, country, journal, etc.) and thematic coding of content to report the data. Frequencies of codes are summarized and presented in graphical form where possible. Microsoft Excel 2010 (Microsoft, Redmond, WA, USA) was used to facilitate descriptive analyses and graphical summaries.

## Results

**Fig. 1:**
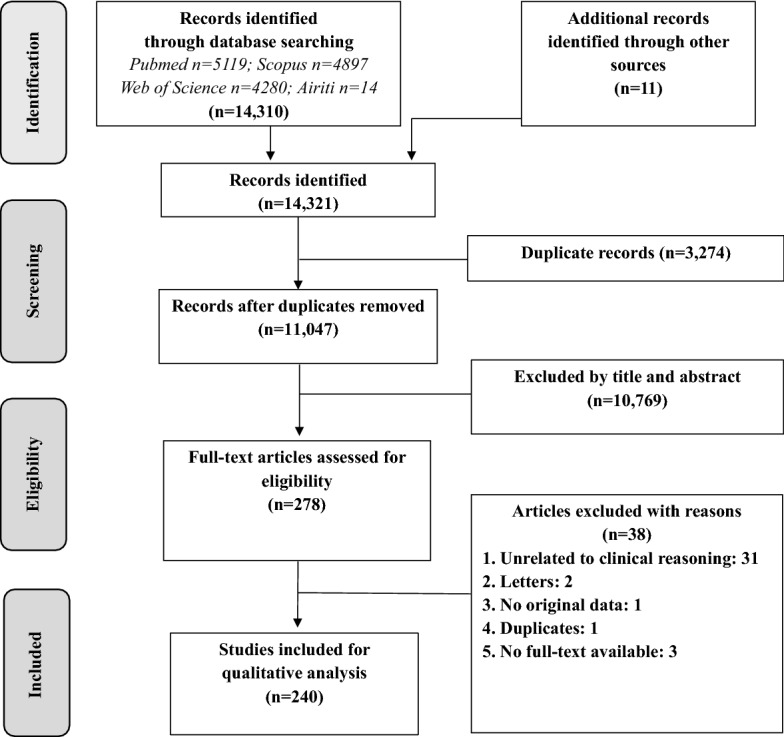
Flowchart of the study selection process

The two separate searches yielded a total of 14,321 potentially relevant articles. After the duplication check and screening, 278 met the eligibility criteria. Following data characterization, 240 articles were included for full coding (Fig. 1).

### Year, Country, Participants and Methodology (RQs 1–4)

**Fig. 2:**
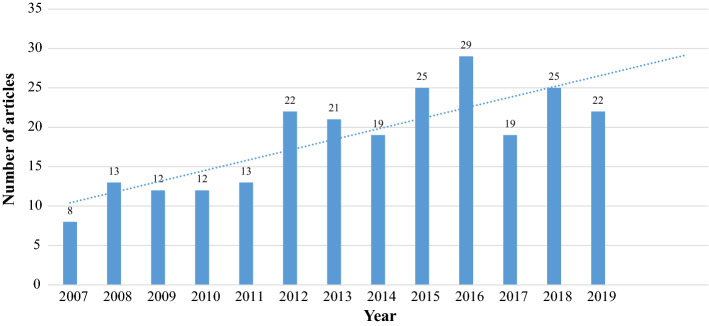
The number of articles on clinical reasoning in Asia between 2007 and 2019

Figure 2 illustrates an increase in number of published articles annually between 2007 and 2019. As we can see, particularly from 2012 (9%, n = 22), there is a steady climb in the number of studies published until 2016 (12%, n = 29) when publications reduce slightly between 2017 and 2019. Over 60% of articles were published by researchers from five countries: South Korea (19%, n = 46), Iran (17%, n = 41), China (15%, n = 36), Taiwan (9%, n = 22) and Turkey (8%, n = 19), see Supplement Table 3. Nursing and medical students were the most common study participants (42%, n = 105 and 34%, n = 86, respectively; see online supplement Table 4). Quantitative approaches (72%, n = 173) were the most prevalent type with an increasing trend, followed by qualitative (20%, n = 49), while mixed-methods (8%, n = 18) approaches were the least common (Supplement Fig. 1).

### The main focus of the published studies (Study themes: RQ5)

We categorized the articles into seven content themes see Table 1 (and online supplement Table 5) based on the frequency in our data, namely: (1) Evaluation of existing courses; (2) Research into critical thinking; (3) Research into decision making; (4) Evaluation of assessment; (5) Research into clinical reasoning; (6) Development of teaching; and (7) Description of concepts, teaching or learning. These content themes contained a variety of sub-themes that came together within each category. We also used coding frequency to support the key issues identified within our content themes (rather than cherry-picking). However, we chose not to report specific numbers of these sub-themes within the themes as they will be relatively low and therefore adding little to our overall understanding (Monrouxe & Rees, 2020).

**Table 1:** Themes identified based on definitions and their respective proportion of articles

Themes	Definitions	Number of studies (%)
(1) Evaluation of existing courses	Measuring the educational outcomes, or teaching process of existing courses	73 (30)
(2) Research into critical thinking	Critical thinking as the focus of research including teaching or learning methods, personal characteristics or assessments related specifically to critical thinking	71 (30)
(3) Research into decision making	Decision making as the focus of research and its relation to clinical disposition, status of clinical competence, teaching or learning methods, or team-based educational programs	28 (12)
(4) Evaluation of assessment	Assessment tools as the focus of research. This includes the guidelines for assessments of clinical reasoning, diagnostic reasoning and clinical judgment, the development process and feedback from participants of an assessment tool	26 (11)
(5) Research into clinical reasoning	Clinical reasoning as the focus of research and its relation to practice methods, learning methods, clinical skills, or personal characteristics	15 (6)
(6) Development of teaching	Any content including critical thinking with original data or development of a new teaching tool without any actual outcome for clinical reasoning. Investigation of new teaching method, use of new teaching method modifications to solve or improve participants’ clinical reasoning or critical thinking disposition, as well as the core competence of teaching with clinical reasoning or critical thinking	12 (5)
(7) Description of concepts, teaching or learning	Any studies involving only the description of a new learning or assessment tool design. Alternatively, the historical systematic review of clinical reasoning or the concept itself	10 (4)

#### Evaluation of existing courses

This category has the highest number of articles coded to it (30%, n = 73), including studies examining the effects of teaching practices (e.g. problem-based learning, high-fidelity simulation) on critical thinking and decision making skills (Agha, 2019; Ahn & Kim, 2015; Lee, Kim, & Park, 2015b, Lee, Lee, Lee, & Bae, 2016; Okubo et al., 2012; Tayyeb, 2013; Yoo, Cho, & Kim, 2019; Yu, Zhang, Xu, Wu, & Wang, 2013; Zarifsanaiey, Amini, & Saadat, 2016). Some articles report comparative studies, for example, suggesting that problem-based learning approaches leads to greater improvement in critical thinking skills than traditional lectures (Choi, Lindquist, & Song, 2014; Gholami et al., 2016; Zhang et al., 2012).

#### Research into critical thinking

This second category comprises articles relating to the assessment of critical thinking (30%, n = 71). Common assessments are the California Critical Thinking Disposition Inventory (and its Chinese version), the California Critical Thinking Skills, Critical Thinking Disposition Inventory (and its Chinese version), Critical Thinking Disposition Scale-Korean version and Watson–Glaser Critical Thinking Appraisal (Atay & Karabacak, 2012; Chen et al., 2010; Huang, Chen, Yeh, & Chung, 2012; Kaya & Yalniz, 2012; Kim, Moon, Kim, Kim, & Lee, 2014; Moattari, Soleimani, Moghaddam, & Mehbodi, 2014; Pai, Eng, & Ko, 2013; Pilevarzadeh, Mashayekhi, Faramarzpoor, & Beigzade, 2014; Suliman & Halabi, 2007; Woo & Tak, 2015; Zhang et al., 2017). Among the subscales of these instrument tools, low scores of truth-seeking, confidence, systematicity and inference are frequently reported in Chinese, Taiwanese, Korean, Turkish, Iranian and Pakistani medical and nursing students (Atay & Karabacak, 2012; Chen et al., 2010; Huang et al., 2012; Kaya & Yalniz, 2012; Kim et al., 2014; Mahmoodabad, Nadrian, & Nahangi, 2012; Moattari et al., 2014; Pai et al., 2013; Penjvini & Hejrani, 2015; Wong, 2007; Woo & Tak, 2015; Yeh, Chen, & Huang, 2009; Yildirim, Ozkahraman, Korkmaz, & Ersoy, 2011; Zhang & Lambert, 2008; Zhang, Li, Wu, & Chen, 2009; Zia & Dar, 2019).

#### Clinical decision making

Articles in this category (12%, n = 28) tend to focus on strategies to develop decision making skills, such as pattern recognition, the implementation of evidence-based medicine or multidisciplinary educational programmes (Hachesu et al., 2012; Ladak et al., 2013; Lee et al., 2009; Marcelo et al., 2013; Phua, See, Khalizah, Low, & Lim, 2012; Ramezani-Badr, Nasrabadi, Yekta, & Taleghani, 2009). One study in particular indicates that preference of cognitive approaches for clinical decision making may be subject to factors, such as gender, specialty, years of practice or training background of the physician, despite both rational and experiential techniques are used in clinical decision making process (Alshaalan, Alharbi, & Alattas, 2019).

#### Evaluation of assessment

Articles coded to this theme (11%, n = 26) primarily report studies that evaluate competence levels in clinical reasoning using Objective Structured Clinical Examinations (OSCE) or Script Concordance Tests (SCT) (Abdelkhalek, Hussein, Sulaiman, & Hamdy, 2009; Iravani, Amini, Doostkam, & Dehbozorgian, 2016; Park, Kang, Lee, & Myung, 2015; See, Tan, & Lim, 2014; Tsai et al., 2008). The SCT is the most common assessment in studies and is considered to be a reliable and validated tool to evaluate clinical reasoning (Iravani et al., 2016; Nazim et al., 2019; Sadeghi et al., 2019; See et al., 2014; Tan, Tan, Kandiah, Samarasekera, & Ponnamperuma, 2014). The SCT is able to differentiate between participants of varying levels of competence and utilized in different clinical disciplines including urology, psychiatry, otolaryngology and neurology (Iravani et al., 2016; Kazour, Richa, Zoghbi, El-Hage, & Haddad, 2017; Nazim et al., 2019; Tan et al., 2014).

#### Clinical reasoning

This theme comprises articles focusing on methods or influential factors for the learning of clinical reasoning (6%, n = 15). For example, two studies demonstrate the effectiveness of learning approaches (cognitive-mapping or illness scripts) in improving medical students’ reasoning performance (Lee et al., 2010; Wu, Wang, Grotzer, Liu, & Johnson, 2016). The use of Summarize history and physical findings, Narrow the differential, Analyze the differential, Probe preceptor about uncertainties, Plan management, and Select case-related issues for self-study, the SNAPPS is considered another approach for promoting clinical reasoning skills in residents and medical students (Kapoor, Kapoor, Kalraiya, & Longia, 2017; Sawanyawisuth, Schwartz, Wolpaw, & Bordage, 2015). In addition, two studies show the positive impact of problem-solving workshop or tutorial on developing clinical reasoning skills in medical students (Matinpour et al., 2014; Rehan, Farooqi, Khan, & Rehman, 2017). Factors associated with clinical reasoning are examined in two further studies: one concludes that it is the culture within the classroom, or the clinical environment, that is likely to exert an influence on medical students’ clinical reasoning through the hidden curriculum (Vidyarthi, Kamei, Chan, Goh, & Lek, 2015); while the other study determines emotional intelligence as one variable that can be used for clinical reasoning prediction (Ashoorion, Liaghatdar, & Adibi, 2012).

#### Development of teaching

Articles coded to this theme (5%, n = 12) primarily focus on investigating new teaching methods for clinical reasoning, decision making or critical thinking. For example, after the implementation of a summary statement practice for teaching clinical reasoning, an idea adopted from Western clinical training, one Japanese study observed the improvement of clinical reasoning skills in resident physicians (Heist, Kishida, Deshpande, Hamaguchi, & Kobayashi, 2016). A mnemonic checklist (TWED, T = threat, W = what else, E = evidence and D = dispositional factors) is introduced as an innovation created to reduce cognitive bias to enable medical students make better quality of clinical decisions (Chew, Durning, & van Merrienboer, Chew, Durning, & van Merrienboer, 2016). Some studies present a new teaching method that involves common teaching models combined with another intervention. For improving various clinical skills in nursing or medical students, these new methods include the development of a simulation scenario and evaluation checklist, the integration of the virtual patient system with the conventional OSCE, the incorporation of social media into problem-based training, and the use of group discussion, simulation and problem-solving interventions in a blended training program (Awan, Awan, Alshawwa, Tekian, & Park, 2018; Lee, Jeong, Kang, Kim, & Lee, 2015a; Lin et al., 2013; Parandavar, Rezaee, Mosallanejad, & Mosallanejad, 2019).

#### Description of concepts, teaching or learning

Articles coded to this category (4%, n = 10) comprise mainly descriptive studies. For example, the effectiveness of problem-based learning on critical thinking and the importance of integrating this learning approach with applications such as portfolios or internet technologies are frequently discussed (Azer, 2008; Chan, 2013; Fan, Jiang, Shi, Wang, & Li, 2018; Jia, Zeng, & Zhang, 2018; Kong, Qin, Zhou, Mou, & Gao, 2014).

### Studies with comparative topics in relation to cultural issues (RQ6)

There are 7 articles (Table 2) which include comparative topics or descriptions in relation to cultural issues (Chan, 2013; Chiang & Chan, 2014; Findyartini et al., 2016; Salsali, Tajvidi, & Ghiyasvandian, 2013; Sawanyawisuth et al., 2015; Sommers, 2018; Wang, Chien, & Twinn, 2012). Of these, one study compared clinical reasoning teaching and learning in Australia with that in Indonesia (Findyartini et al., 2016). This study found that Indonesian students score lower on the Flexibility subscale of the Diagnostic Thinking Inventory than their Australian counterparts. Focus group interview data from this study also suggested a tendency toward uncertainty avoidance in students from the Indonesian context (Findyartini et al., 2016).

**Table 2:** Overview of 7 studies with comparative-related topics or cultural implications

1st Author Year; Country; Study Design	Participants	Assessment	Comparative topics or Cultural implications	Major findings
Sommers (2018) Indonesia Review	53 published articles	Literature review	Tools for measuring critical thinking (CT), clinical reasoning (CR), and/or clinical judgment in nursing students from diverse cultures The relationship between culture and learning	Most studies were conducted in Western countries CT tools from Western countries were directly translated without cultural adaptation, frequently resulted in lower measurements in non-Western countries Students from culturally diverse backgrounds have different perspectives, ways of learning, and ways of processing information Little is known about how culture affects teaching–learning relationship in nursing students
Findyartini (2016) Indonesia Mixed	166 (Australian) and 203 (Indonesian) medical students 45 students (Indonesia, 31; Australia, 14) in focused groups and 24 medical teachers (Indonesia, 11; Australia, 13) in individual interviews	Diagnostic Thinking Inventory (DTI) Focused group discussions and individual interviews	Teaching and learning of CR between universities in Australia and Indonesia	Indonesian students scored lower on the Flexibility in thinking subscale of the DTI A cultural tendency toward uncertainty avoidance is found in the Indonesian context, along with a preference for standardized CR processes and seeking complete information Cultural differences in attitudes to authoritative sources and attitudes to uncertainty between the Indonesian and the Australian medical students may influence conceptualization, teaching and learning of CR in each setting
Sawanyawisuth (2015) Thailand Quantitative	207 fifth-year medical students in Thailand	Case presentations	Outcomes related to expressing CR and uncertainties	Thai medical students expressed uncertainties far less often than their American counterpart from the original study by Wolpaw et al. (2009) Cultural differences of passive nature in Thai students leads to reluctance to express uncertainties during case presentations
Chiang VCL (2014) Hong Kong Mixed	132 undergraduate pre-registration nursing students in Hong Kong	The California Critical Thinking Disposition Inventory (CCTDI) Focus group interviews	The percentages of CCTDI sub-scores were compared with a sample of 267 representative sample of undergraduates from different universities around the US and Canada (Facione & Facione, 2007)	The CCTDI scores in these Hong Kong nursing students were all unfavorable comparing with the exemplar students from the US and Canada Cultural differences and their possible relationships to the practice and experience of teaching and learning between Hong Kong and Western countries might have contributed to the differences in CT disposition
Salsali (2013) Iran Review	795 articles published in English and Persian language	CCTDI scores were reviewed and compared	Nursing students’ CT dispositions in Asian and non-Asian countries	Asian nursing students had lower CT scores than non-Asian students possibly due to cultural differences
Chan (2013) Iran Systematic Review	17 published articles	Systematic Review	How CT is perceived and influential factors of learning and teaching of CT in nursing education	Only two studies explored Asian educators' perspectives in CT Cultural background may either hinder or facilitate CT Students in some countries who try to avoid conflicts do not question teachers (Jenkins, 2011; Kawashima, 2003; Mangena & Chabeli, 2005)
Wang (2012); China; Qualitative	12 baccalaureate-prepared registered nurses from a teaching hospital in Tianjin, China	Semi-structured interviews	Perspectives of and perceived autonomy in clinical decision making (CDM)	Chinese nurses in this study revealed lower level of autonomy in CDM, when compared with the nurses in developed Western countries (Bradley & Nolan, 2007; Cajulis & Fitzpatrick, 2007; Carryer, Gardner, Dunn, & Gardner, 2007; Kleinpell-Nowell, 2001; Stenner & Courtenay, 2008) Socio-cultural beliefs relating to nursing in Chinese society may have an impact on nurses’ CDM autonomy in clinical practice

*CCTDI* the California critical thinking disposition inventory, *CDM* clinical decision-making, *CR* clinical reasoning, *CT* critical thinking, *DTI* diagnostic thinking inventory

One study from Thailand applied the learning approach of clinical reasoning SNAPPS derived from a Western culture to students at the Thailand University (Sawanyawisuth et al., 2015). The authors from this study suggested that the cultural differences in terms of Thai students’ passive nature may result in expression of uncertainties far less often than their American counterparts during case presentations (Wolpaw, Papp, & Bordage, 2009). Two further studies conducted in Hong Kong and China, respectively also compared the critical thinking disposition or clinical decision making between their local nursing participants and their counterparts in the studies from other Western countries (Chiang & Chan, 2014; Wang et al., 2012). By comparison to their Western counterparts, the Hong Kong nursing students had lower critical thinking scores (Chiang & Chan, 2014) while the Chinese registered nurses showed a lower level of autonomy in decision making (Wang et al., 2012).

Two literature reviews (Salsali et al., 2013; Sommers, 2018) and one systematic review (Chan, 2013) examined critical thinking dispositions in nursing education. Salsali et al. (2013) addressed differences in critical thinking dispositions between Asian and Non-Asian nursing students. Two other reviews discussed the influence of different cultural backgrounds on teaching or learning of critical thinking (Chan, 2013; Sommers, 2018). In the systematic review by Chan (2013), the author suggests that the cultural background of learners and educators plays an important role in learning and teaching critical thinking. Considering the finding that most studies included in Chan’s review were conducted in Western countries, there is also an emphasis on the need for more studies exploring cultural diversity in critical thinking or clinical reasoning (Chan, 2013).

### Journals, publication language and funding (RQs 7 & 8)

**Fig. 3:**
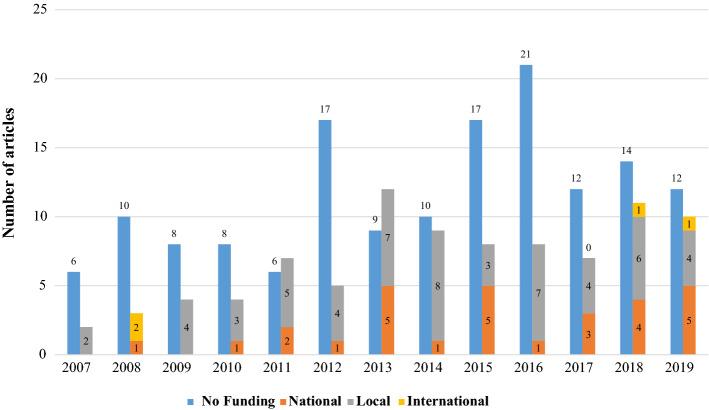
The funding status of clinical reasoning articles

English (91%, n = 219) was the most frequently identified language (Supplement Fig. 2), followed by traditional and simplified Chinese (6%, n = 14), and Korean (3%, n = 7). The majority of articles were published in Nurse Education Today, followed by BMC Medical Education (Supplement Fig. 3). The majority of published articles reported unfunded studies (63%, n = 150: Fig. 3); when studies were funded, this was mainly by local institutions (63% of funded studies, n = 57), but some were nationally funded (32%, n = 29) with the minority being internationally funded (4%, n = 4).

## Discussion

Our scoping review of clinical reasoning research across Asia has revealed a number of trends. First of all, the number of studies on clinical reasoning in Asia has increased in both quantity and quality. The funding of studies (mainly institutional but also national) also shows an increase around 2011–2014, although the meaning of why this is the case is unclear. However, international collaboration (and funding) was found to be infrequent and fewer articles are published in non-English languages over the course of the decade. The most frequent topic and target population identified respectively are nursing and nursing students, followed by medical education and medical students. Also, the principal research theme is the evaluation of existing courses, focusing specifically on problem-based learning. Evaluation of assessment is rarely studied in the context of clinical reasoning in Asian countries, as is the process of clinical reasoning and decision making itself. Further, limited attention is placed on the development of research methodology and theory.

Indeed, our findings concur with a recent systematic scoping review of the healthcare professions education research literature from Taiwan (Monrouxe et al., 2020), in which the authors revealed a growing publication trend of healthcare professions education research over twelve years (2006–2017). Furthermore, they also found low international research collaboration, consistent with our study (as reported for RQ6) (Monrouxe et al., 2020). This suggests that the collaborative effort between Western and Eastern international partners is an area to be fostered and strengthened, with a focus on developing more multi-national, cross-cultural research on clinical reasoning. In the context of a body of cognitive research on clinical reasoning that has been predominately undertaken in a single (Western) culture, and given the plethora of research that suggests clear differences in thinking and reasoning between Eastern and Western cultures (Choi et al., 2007, 2008; Li et al., 2018; Nisbett & Miyamoto, 2005), it appears that knowledge around clinical reasoning is particularly biased towards Western cognitive practices. Leveraging various research and development funds that are available in the Asian region (Monrouxe et al., 2020) could be a promising strategy towards developing such work.

We found only a few comparative studies cross-culturally (n = 7). More specifically, there is only one study that actually compares the clinical reasoning in medical students between Asian and Western cultures (Findyartini et al., 2016). The remaining studies are reviews, or those conducted mainly with nursing participants that utilize the exemplary results from Western countries to compare critical thinking with their local findings (Chan, 2013; Chiang & Chan, 2014; Salsali et al., 2013; Sawanyawisuth et al., 2015; Sommers, 2018; Wang et al., 2012). The Asian participants in these studies were often associated with unfavorable critical thinking scores or characteristics considered to be typically found in Asian cultures such as subordination to authority or uncertainty avoidance. This cultural tendency toward uncertainty avoidance and seeking complete information in the Asian context reflects the work by Nisbett and Miyamoto (2005) where Holistic thinkers (representing Eastern cultures) tend to seek a compromise between conflicting pieces of information and consider many aspects of information before coming to a decision (Choi et al., 2003, 2007, 2008; Levett-Jones et al., 2010). While none of the studies relate their findings to the those by Nisbett or Choi, all the authors attributed the differences in their findings to cultural influences with evidence from other literature on culture in their discussion sections.

There has been a paradigm shift in healthcare professions education research from being heavily scientific-oriented to having more inclusion of healthcare professions education research from across the social sciences (Hong et al., 2010; Monrouxe & Rees, 2009; Schuwirth & van der Vleuten, 2006). That healthcare professions education research more generally has gained in popularity over the years may also explain our finding of increasing trend of research on clinical reasoning in Asia (Eva, 2005; Hong et al., 2010; Monrouxe et al., 2020; Schuwirth & van der Vleuten, 2006). In addition, this might be an indicator for increased awareness among Asian healthcare professions educators on the importance of clinical reasoning in the training of healthcare professionals.

Although we have reviewed the literature on clinical reasoning across Asia, our analysis also raises questions about aggregating Asian countries as one big ‘Asian Culture’. Since Asia is a large continent with very diverse cultures, the question arises, to what extent can the whole of Asia be taken as ‘One Culture’? Approximately 68% (n = 164) of the articles in our study come from five Asian countries (South Korea, Iran, China, Taiwan and Turkey), while the remaining 32% originate from another 16 countries. It might be that countries in Asia are better grouped according to them possessing a more or less similar culture with one another, rather than together (Hoftede, Hofstede, & Minkov, 2010).

It is interesting to note that the most prominent studied area in clinical reasoning is related to the evaluation of existing courses and critical thinking, with the least prominent being related to the development and description of concepts, teaching or learning. This suggests that current research focuses more on the learning of clinical reasoning, rather than the fundamental process of clinical reasoning itself. Such a focus suggests that there might be a tendency to accept prevailing Western understandings of how healthcare professionals think and reason clinically as being how healthcare professionals across the world—irrespective of their cultural upbringing. This is despite there being evidence that suggests this might not be the case.

### Challenges and strengths

The lack of critical appraisal, or possibility of missing relevant studies, has been reported as a key challenge in scoping reviews (Brien, Lorenzetti, Lewis, Kennedy, & Ghali, 2010; Feehan, Beck, Harris, MacIntyre, & Li, 2011; Hand & Letts, 2009). However, scoping reviews are not intended to be exhaustive (Boydell, Gladstone, Volpe, Allemang, & Stasiulis, 2012; Cameron, Tsoi, & Marsella, 2008; Levac et al., 2010). We also considered the fuzzy boundaries between the terminologies used to be a limitation (Young et al., 2018). For example, some researchers used the term clinical reason, while others used the term critical thinking, both of which have overlapping elements and sometimes used interchangeably. Although we used authors’ terminology to drive our coding, this does not overcome the core issue of this poorly integrated body of literature. Another limitation is that concepts similar to clinical reasoning may be described using different terms in local languages. This leads to the potential bias of the lack of sufficient locally-published articles in non-English journals in this review. However, due to the range of Asian collaborators on this study, we were able to access some non-English but relevant articles, thus the bias of this issue has been minimized to the lowest extent possible.

### Implications for research

To our knowledge, this is the first scoping review study exploring the current status of clinical reasoning research across Asia. However, we were unable to identify cultural differences in the process of clinical reasoning between East Asian and Western cultures, despite indirect evidence of existing distinct critical thinking dispositions between these two groups. We would argue that it is important to promote fundamental research in clinical reasoning across different cultural contexts in order to fully understand the underlying mechanism of healthcare professionals’ clinical reasoning processes. These fundamental results will inform researchers and educators on the best way to teach, learn, evaluate and assess the clinical reasoning abilities within different cultural environments. Furthermore, such research might consider comparing groups of countries based on their distinct cultural similarities, rather than having a “one Asia” approach. One potential way forward for this would be to utilise Hofstede’s classification of national cultures (Hoftede et al., 2010). A deeper analysis in future work might reveal interesting findings about differences in thinking and clinical reasoning patterns *within* Asia as well as outwith. A better understanding of the healthcare education systems in a culturally sensitive manner can therefore provide Asian healthcare professionals with adequate clinical reasoning curricula tailored appropriately for the Asian learning environment.

## Supplementary Information

Below is the link to the electronic supplementary material.

Supplementary file1 (DOCX 127 KB)
