# Supplementary material for: A scoping review of clinical reasoning research with Asian healthcare professionals
Source: Adv Health Sci Educ Theory Pract. 2021 Jul 12;26(5):1555–79. doi: 10.1007/s10459-021-10060-z (PMC8610955; doi:10.1007/s10459-021-10060-z)
Supplement: Supplementary file 1 — Supplementary file1 (DOCX 127 KB) [file 10459_2021_10060_MOESM1_ESM.docx]

## **Supplementary materials**

### Figure 1. Methodology used for clinical reasoning articles

### Figure 2. The number of articles each year by language

### Figure 3. The top five journals publishing clinical reasoning articles from Asia

Table 1. Search strategy

| 1. clinical reasoning |
| --- |
| 2. thinking process |
| 3. differential diagnosis |
| 4. decision making |
| 5. problem-based learning |
| 6. critical thinking |
| 7. health care profession |
| 8. institution |
| 9. medical students |
| 10. nursing students |
| 11. trainee |
| 12. residents |
| 13. or/1-7 |
| 14. or/8-13 |
| 15. 14 and 15 |
| 16. limit 15 to yr=”2007-2019” |

### Table 2. Inclusion/Exclusion criteria

| **Inclusion Criteria** | **Exclusion Criteria** |
| --- | --- |
| - Peer-reviewed full journal articles - Original data - From 2007-2019 - Publications from Asian countries - Full-text available - Focuses on clinical reasoning (or its derivatives) | - Conference recordings, letters or editorials, non-peer-reviewed articles - Articles related to clinical reasoning but has no Asian data or authors - Content lacks original data or discussion on clinical reasoning |

### Table 3. The proportion of articles on clinical reasoning by country

| **Country** | **Number of studies (%)** | |
| --- | --- | --- |
| South Korea | 46 (19) |  |
| Iran | 41 (17) |  |
| China | 36 (15) |  |
| Taiwan | 22 (9) |  |
| Turkey | 19 (8) |  |
| Japan | 11 (5) |  |
| Singapore; Hong Kong | 10 (4)* |  |
| Saudi Arabia | 8 (3) |  |
| Pakistan | 7 (3) |  |
| Malaysia | 5 (2) |  |
| India | 4 (2) |  |
| Indonesia | 3 (1) |  |
| Israel | 3 (1) |  |
| Lebanon | 3 (1) |  |
| Thailand | 3 (1) |  |
| United Arab Emirates | 3 (1) |  |
| Qatar | 2 (1) |  |
| Bahrain | 1 (0) |  |
| Jordan | 1 (0) |  |
| Oman | 1 (0) |  |
| Philippines | 1 (0) |  |
| Sri Lanka | 1 (0) |  |

Table 4. Types of study population identified for articles on clinical reasoning in Asia between 2007-2019

|  | 2007 | 2008 | 2009 | 2010 | 2011 | 2012 | 2013 | 2014 | 2015 | 2016 | 2017 | 2018 | 2019 | TOTAL (%) |
| --- | --- | --- | --- | --- | --- | --- | --- | --- | --- | --- | --- | --- | --- | --- |
| Nursing Students | 4 | 6 | 5 | 5 | 7 | 7 | 9 | 12 | 15 | 10 | 8 | 10 | 7 | 105 (42) |
| Medical Students | 4 | 5 | 4 | 5 | 3 | 8 | 9 | 2 | 7 | 9 | 7 | 11 | 12 | 86 (34) |
| Residents | 0 | 1 | 1 | 1 | 1 | 3 | 1 | 3 | 1 | 6 | 1 | 3 | 4 | 26 (10) |
| Nurses | 0 | 0 | 1 | 2 | 2 | 3 | 0 | 1 | 2 | 2 | 1 | 0 | 0 | 14 (6) |
| Experts | 0 | 0 | 0 | 0 | 0 | 0 | 0 | 1 | 1 | 1 | 0 | 0 | 1 | 4 (2) |
| Teachers | 2 | 0 | 0 | 0 | 0 | 0 | 0 | 0 | 0 | 0 | 0 | 1 | 0 | 3 (1) |
| Interns | 0 | 0 | 0 | 0 | 0 | 0 | 0 | 1 | 0 | 0 | 1 | 0 | 1 | 3 (1) |
| Trainees | 0 | 0 | 0 | 0 | 0 | 0 | 0 | 0 | 0 | 1 | 1 | 1 | 0 | 3 (1) |

Table 5. Content themes for articles on clinical reasoning in Asia between 2007-2019.

|  | 2007 | 2008 | 2009 | 2010 | 2011 | 2012 | 2013 | 2014 | 2015 | 2016 | 2017 | 2018 | 2019 | Total (%) |
| --- | --- | --- | --- | --- | --- | --- | --- | --- | --- | --- | --- | --- | --- | --- |
| Evaluation of existing courses | 2 | 6 | 2 | 3 | 4 | 5 | 9 | 5 | 5 | 14 | 5 | 4 | 9 | 73 (30) |
| Research into critical thinking | 2 | 3 | 6 | 5 | 4 | 8 | 5 | 7 | 8 | 0 | 6 | 10 | 7 | 71 (30) |
| Research into decision making | 1 | 2 | 3 | 2 | 1 | 7 | 4 | 1 | 2 | 5 | 1 | 3 | 1 | 28 (12) |
| Evaluation of assessment | 0 | 1 | 1 | 0 | 1 | 1 | 1 | 4 | 6 | 2 | 3 | 2 | 4 | 26 (11) |
| Research into clinical reasoning | 0 | 0 | 0 | 1 | 2 | 1 | 0 | 1 | 2 | 5 | 2 | 1 | 0 | 15  (6) |
| Development of teaching | 2 | 1 | 0 | 0 | 0 | 0 | 1 | 0 | 2 | 3 | 1 | 1 | 1 | 12 (5) |
| Description of concepts, teaching or learning | 1 | 2 | 0 | 0 | 0 | 0 | 1 | 1 | 0 | 0 | 1 | 4 | 0 | 10  (4) |
